# Evaluation of the Effects of Sedation and Anesthesia on Total Lung Volume and Attenuation in Rabbit Lung CT Exams

**DOI:** 10.3390/ani14233473

**Published:** 2024-12-01

**Authors:** Roberto Sargo, Inês Tomé, Filipe Silva, Mário Ginja

**Affiliations:** 1 Veterinary Teaching Hospital, University of Trás-os-Montes e Alto Douro, 5000-801 Vila Real, Portugal; inestome@utad.pt (I.T.); fsilva@utad.pt (F.S.); mginja@utad.pt (M.G.); 2 CECAV-Veterinary and Animal Science Research Centre and AL4Animals—Associate Laboratory for Animal and Veterinary Science, University of Trás-os-Montes e Alto Douro, Quinta de Prados, 5000-801 Vila Real, Portugal; 3 Department of Veterinary Science, University of Trás-os-Montes e Alto Douro, 5000-801 Vila Real, Portugal

**Keywords:** rabbit, computed tomography, lung volume, lung attenuation, sedation

## Abstract

Respiratory disease is common in rabbits, but subclinical conditions can be challenging to diagnose and may cause respiratory problems during anesthesia. CT is the preferred method for diagnosing lung diseases, but anesthesia can alter lung volume and cause lung lobe collapse. In this study, seventeen healthy 5-month-old male New Zealand white rabbits underwent thoracic CT scans under different conditions. Rabbits were sedated with midazolam and butorphanol and scanned in a sphinx position; they were then anesthetized with dexmedetomidine and ketamine and scanned again in sternal recumbency during spontaneous breathing. Lastly, apnea was induced using intermittent positive pressure ventilation (IPPV) for a final scan. Lung volume and density were measured using the 3D Slicer version 5.6.2 software, with thresholds set between −1050 and −100 Hounsfield Units (HU). Sedated animals had significantly higher total lung volume (69.39 ± 10.04 cm^3^) than anesthetized (47.10 ± 9.28 cm^3^) and anesthetized apnea rabbits (48.60 ± 7.40 cm^3^). Mean lung attenuation during sedation was −611.26 HU (right) and −636.00 HU (left). After anesthesia induction, values increased to −552.75 HU (right) and −561.90 HU (left). Following apnea induction, attenuation slightly decreased to −569.40 HU (right) and −579.94 HU (left). The results indicate that sedation may be preferable for rabbit lung CT to minimize anesthesia-related changes.

## Simple Summary

Rabbits are commonly affected by respiratory diseases. Their small size and high respiratory rates make diagnosing these conditions difficult. Computed tomography (CT) is considered the best imaging modality for diagnosing lung problems in rabbits; however, it requires anesthesia for proper positioning and to minimize movement. Despite its advantages, there is a notable scarcity of studies investigating the baseline imaging characteristics of normal rabbit lungs. Anesthesia is known to produce artifacts that can exacerbate or obscure abnormal findings in CT images. On the other hand, some animals may not be stable enough upon presentation to undergo anesthesia, which can delay diagnosis. Sedation has been recommended in other species, such as cats and dogs, as an alternative to anesthesia, making CT scans safer for compromised patients. Understanding the effects of sedation and anesthesia on CT images of rabbit lungs is essential for providing better diagnostic options for these patients.

## 1. Introduction

Respiratory diseases are among the most common reasons for rabbits’ visits to veterinary practices. However, the unique anatomy and physiology of the rabbit respiratory system make accurate diagnosis challenging [1,2]. While thoracic radiography is the primary screening tool for lower respiratory tract diseases, it has limitations, particularly in precisely identifying and characterizing lung lesions [3,4].

Thoracic computed tomography (CT) is considered the gold standard for evaluating lung diseases in rabbits, offering superior sensitivity in detecting abnormalities in lung parenchyma [5,6]. Despite its advantages, the high cost and limited availability of CT equipment present significant barriers to its widespread use [5,6].

Although CT is regarded as the most reliable diagnostic method for rabbit lung disease, there is only one published study on normal tomographic images of rabbit lungs in which some animals showed pathological changes [7]. The small size of rabbits requires proper positioning to achieve high-quality imaging, making sedation and/or anesthesia essential to ensure accurate results [2,3,4,6]. Unfortunately, respiratory diseases in rabbits are often subclinical, making it difficult to detect them during routine clinical examinations, and they frequently contribute to anesthesia-related complications [2,8].

General anesthesia, although recommended for immobilizing patients, can exacerbate imaging artifacts, particularly due to the more pronounced atelectasis observed in dependent lung regions [9]. Atelectasis has been shown to develop immediately after anesthesia induction [10]. In contrast, sedation minimizes anesthesia-related artifacts on thoracic CT images. Sedation protocols generally result in less muscle relaxation compared to anesthesia, leading to a lower risk of compression atelectasis [11]. Furthermore, animals breathing room air are less likely to develop diffusion atelectasis, which can occur due to the high oxygen fraction delivered during anesthesia [12]. However, prolonged sedation may increase lung attenuation [13], and respiratory motion artifacts may become more pronounced [11].

The objective of this study was to investigate the effects of sedation and anesthesia on total lung volume and mean lung attenuation in healthy rabbits. We hypothesize that anesthesia leads to a reduction in lung volume and an increase in lung attenuation, potentially impairing the accuracy of lung CT evaluations.

## 2. Materials and Methods

### 2.1. Study Design and Subject Inclusion

A prospective study was designed to complement the use of animals involved in a separate study focused on CT imaging of hip joints. All procedures complied with European and national legislation concerning the protection of animals used for scientific purposes (European Directive 2010/63/EU and National Decree-Law 113/2013) and were approved by the relevant Portuguese authority, the Directorate-General for Food and Veterinary (DGAV_0421/000/000/2022). Seventeen healthy male, five-month-old New Zealand White rabbits were used in this study. The sample size was based on a statistical significance level of 0.05, a large effect size of 1.0, and a power of 0.80 using a *t*-test table, which resulted in a minimum sample of 17 observations [14].

The rabbits were housed individually in cages measuring 80 cm × 55 cm × 55 cm, each with an elevated platform inside. The housing area was a ventilated room with a temperature maintained between 19–21 °C and relative humidity levels of 50–60%. The animals were kept under a 12-h light/dark cycle with controlled artificial lighting. They were fed a standard pellet diet and had free access to water, with no fasting protocols applied. On the day of the CT exams, each rabbit underwent a comprehensive physical exam to rule out any signs of respiratory disease, including recording body weight.

### 2.2. Sedation and Anesthesia Protocols and Computed Tomography Image Acquisition

The rabbits were sedated with butorphanol (Butomidor^®^, Richter Pharma AG, Wels, Austria, at 0.4 mg/kg, IM) and midazolam (Dormazolan^®^, Le Vet Beheer B.V., Oudewater, The Netherlands, at 0.5 mg/kg, IM). They were then immediately placed inside a padded cardboard box secured on the CT bed and allowed to acclimate for ten minutes. During this time, their body positions were checked to ensure they remained in the prone position before conducting the first CT scan.

All scans were acquired in a cranial-to-caudal direction using a lung algorithm, with a tube voltage of 100 kVp, a tube current of 80 mA, a slice thickness of 1.25 mm, a rotation time of 0.98 s, and a pitch of 1.38, performed on a 16-slice scanner (Revolution™ ACT, General Electric Medical Systems, Buckinghamshire, UK).

The second CT scan was performed under general anesthesia, with the rabbits breathing spontaneously. Anesthesia was induced using ketamine (Ketamidor^®^, Richter Pharma AG, Wels, Austria, at a dose of 20 mg/kg, intramuscularly) and dexmedetomidine (Sedadex^®^, Le Vet Beheer B.V., Oudewater, The Netherlands, at a dose of 50 μg/kg, intramuscularly). The animals were placed in sternal recumbency, and following induction, tracheal intubation was achieved using a 3 mm uncuffed endotracheal tube. Anesthesia was maintained with 1.5% isoflurane (IsoFlo100%^®^, Zoetis, Oeiras, Portugal) delivered in 100% oxygen at a flow rate of 2 L/minute. Throughout the procedure, the rabbits remained in sternal recumbency with their forelimbs extended forward, heads positioned between them, and hindlimbs flexed in a natural resting posture. For the final CT scan, apnea was induced through positive pressure ventilation, after which the scan was promptly acquired.

All scans were acquired in a cranial-to-caudal direction using a lung algorithm, with a tube voltage of 100 kVp, a tube current of 80 mAs, a slice thickness of 1.25 mm, a rotation time of 0.98 s, and a pitch of 1.38, performed on a 16-slice scanner (Revolution™ ACT, General Electric Medical Systems, Buckinghamshire, UK).

### 2.3. Image Analysis and Segmentation

Image evaluation, measurements, and computer-generated segmentation for all scans were made by a single veterinarian with more than ten years of diagnostic imaging experience in exotic animals (RS). The images were evaluated using a lung window setting (WL: −500, WW: 1400).

This study used a 3D Slicer version 5.6.2 [15], a free, open-source medical software for visualization, processing, segmentation, and image analysis of all acquired scans. The workstation used for this analysis was an HP Envy x360, equipped with an 11th Gen Intel^®^ Core ™ i7-1195G7 @ 2.9 GHz processor, a 512 GB SSD, 16 GB of RAM, and an Intel IRISx^e^ graphics card.

Each scan was imported into the software, where masking techniques were applied using specific Hounsfield unit (HU) thresholds to differentiate anatomical structures and delineate tissue boundaries. The following threshold limits were applied: 350 HU to −100 HU for identifying the thoracic wall, abdominal soft tissues, mediastinal structures, and pulmonary vasculature; −1050 HU to −900 HU for the trachea and primary to tertiary bronchi; and −899 HU to −101 HU for the pulmonary parenchyma [11,16].

**Figure 1:**
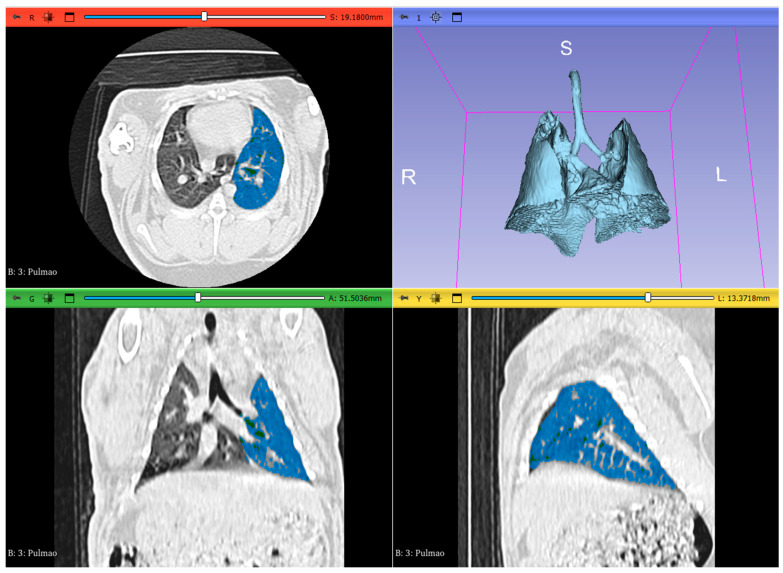
3dSlicer extension LungCTanalyser threshold segmentation of the left lung of a sedated rabbit, selecting inflated aerated tissue (−899 to −500 HU) (transverse, dorsal, and sagittal lung planes; upper left and bottom images, respectively). 3D reconstruction of lung and trachea, obtained from the masked segments in Lung Segmentator extension (upper right corner) (R—right; L—left; S—superior).

A semi-automated approach was used with the Lung CT Segmenter extension, as the AI-powered segmentation could not accurately define the boundaries of the pulmonary parenchyma. The volume was uploaded into LungCTAnalyzer, which used the previous segmentation to differentiate the lungs into different categories: emphysema (−1050 to −900 HU), normal lung tissue (−899 to −500 HU), infiltrated lung/ground-glass opacity (GGO) (−499 to −101 HU), collapsed lung and vessels (−100 to 1000 HU) [11,13] (Figure 1). The software automatically calculated the volume and mean attenuation for each lung and each defined threshold, generating a table with all the values. The volume of each segmented region was quantified in cubic millimeters (mm^3^), and attenuation was in HU. Segmentation was performed for both lungs, including individual lobes, as well as subdividing the lungs into upper and lower halves and dorsal and ventral regions. The centroid of each lung was calculated to facilitate this, allowing for the creation of quadrants to separate the dorsal from the ventral regions and vice versa. A similar methodology was applied to distinguish the upper and lower sections of the lungs. In a second reading session, all measurements were repeated by the same operator (RS) to assess repeatability.

### 2.4. Statistical Analysis

The data collected was recorded using Microsoft Excel for Windows. Statistical analyses were conducted using IBM SPSS Statistics, Version 30.0.0.0 (171). The variables used for the analysis included lung volume (cm^3^), pulmonary index (PI; cm^3^/kg), and lung attenuation (HU). The pulmonary index was calculated by dividing the lung volume by the animal’s body weight in kilograms. Descriptive statistics were applied to each variable, which included calculating the mean, standard deviation, and range.

Linear regression was used to assess the relationship between body weight and total lung volume. To evaluate the repeatability of repeated measurements generated by 3D Slicer for lung volume and lung attenuation (Sessions 1 and 2) and the reproducibility of Sessions 1 measurements from CT data acquired under sedation, anesthesia, and apnea, we used a paired *t*-test and the Intraclass Correlation Coefficient (ICC).

## 3. Results

### 3.1. Study Population

A total of 17 male, 5-month-old New Zealand White rabbits were included in this study, each with a body condition score of 5/9. The body weight ranged from 3.202 to 4.432 kg, with a mean weight of 3.895 ± 0.305 kg. A statistically significant positive regression coefficient between body weight and total lung volume was found in sedated animals (*p* = 0.0027, R^2^ = 0.46). However, no significant correlation was observed in anesthetized animals during spontaneous breathing or after apnea induction. The pulmonary index (PI), or mean total lung volume per kilogram of body weight, was 17.79 ± 1.93 cm^3^/kg for sedated animals.

### 3.2. Lung Volume and Attenuation

Under sedation, the mean total lung volume during Session 1 was 69.39 ± 10.04 cm^3^, with right and left lung mean volumes of 38.82 ± 5.11 cm^3^ and 30.57 ± 5.54 cm^3^, respectively. In Session 2, the mean total lung volume was 69.68 ± 9.89 cm^3^, with right and left lung mean volumes of 38.82 ± 4.95 cm^3^ and 30.97 ± 5.82 cm^3^, respectively. There were no statistically significant differences between sessions (*p* > 0.05, paired *t*-test). The ICC demonstrated strong agreement > 0.97 (*p* < 0.05) (Table 1).

Anesthesia resulted in a significant reduction in lung volume. During Session 1, the mean total lung volume was 47.10 ± 9.28 cm^3^, with the right lung mean volume of 27.82 ± 5.40 cm^3^ and the left lung with 19.28 ± 4.41 cm^3^. In Session 2, the mean total lung volume was 47.29 ± 9.60 cm^3^, with individual mean lung volumes of 27.70 ± 5.62 cm^3^ for the right lung and 19.58 ± 4.56 cm^3^ for the left lung, showing no statistically significant differences (*p* > 0.05, paired *t*-test). The ICC demonstrated strong agreement > 0.97 (*p* < 0.05) (Table 2).

During apnea, the mean total lung volume in Session 1 was 48.60 ± 7.40 cm^3^, with right and left lung mean volumes measuring 28.26 ± 3.88 cm^3^ and 20.34 ± 4.36 cm^3^, respectively. In Session 2, the mean total lung volume was 48.35 ± 7.81 cm^3^, with the right lung mean volume measuring 28.22 ± 4.56 cm^3^ and the left lung mean volume measuring 20.13 ± 4.11 cm^3^. The two sessions had no statistically significant differences (*p* > 0.05, paired *t*-test). The ICC demonstrated strong agreement > 0.91 (*p* < 0.05) (Table 3).

The repeatability of attenuation measurements was assessed across the two sessions. In Session 1, the mean attenuation for the aerated region of the right lung (ranging from −899 to −500 HU) was −675.08 ± 18.23 HU, while the left lung showed a mean attenuation of −690.03 ± 14.19 HU in sedated animals. In Session 2, the values were −675.21 ± 18.36 HU for the right lung and −689.96 ± 13.96 HU for the left lung. There were no statistically significant differences between the two sessions (*p* > 0.05, paired *t*-test). The ICC demonstrated strong agreement > 0.99 (*p* < 0.05) (Table 1).

During anesthesia in Session 1, the mean attenuation for the aerated area of the right lung was measured at −633.47 ± 36.15 HU, and for the left lung, it was −632.24 ± 38.75 HU. In Session 2, the mean attenuation values were −632.73 ± 38.75 HU for the right lung and −631.20 ± 39.63 HU for the left lung, with no significant differences observed between the sessions (*p* > 0.05, paired *t*-test). The ICC demonstrated strong agreement > 0.99 (*p* < 0.05) (Table 2).

Following the induction of apnea in Session 1, the mean attenuation for the aerated right lung was −627.23 ± 33.98 HU, while for the left lung, it was −632.77 ± 38.28 HU. In Session 2, the mean values were similar, with −627.05 ± 33.97 HU for the right lung and −632.23 ± 38.59 HU for the left lung. Again, no statistically significant differences were found between sessions (*p* > 0.05, paired *t*-test). The ICC demonstrated strong agreement > 0.99 (*p* < 0.05) (Table 3).

With a strong agreement between the data collected on both reading sessions, Session 1 data was used for the reproducibility analysis. A paired *t*-test indicated highly statistically significant differences in total lung volumes, as well as in right and left lung volumes, when comparing rabbits under sedation with those under anesthesia during spontaneous breathing and after apnea induction (*p* < 0.001) (Table 1, Table 2 and Table 3). However, no significant differences were observed in total lung volume (*p* = 0.326), right lung volume (*p* = 0.672), or left lung volume (*p* = 0.105) when comparing measurements taken under anesthesia with those taken after apnea induction (Table 2 and Table 3).

The evaluation of inflated lung volumes in the dorsal and ventral regions was performed using regional segmentation data (−899 to −500 HU). Descriptive statistics were applied, and the results are summarized in Table 4. As anticipated, the dependent regions (ventral part) of the lungs showed a greater reduction in volumes after anesthesia. In the ventral regions, the mean reduction in lung volume was 50.26% for the left lung and 48.97% for the right lung. The dorsal regions also exhibited a notable reduction, with inflated volumes decreasing by 35.91% for the left lung and 34.25% for the right lung.

The mean lung attenuation during sedation was measured as −611.26 ± 35.01 HU for the right lung and −636.00 ± 30.67 HU for the left lung. Following anesthesia induction, the mean lung attenuation increased to −552.75 ± 47.76 HU and −561.90 ± 53.41 HU for the right and left lungs, respectively. After inducing apnea, there was a slight decrease in lung attenuation, with the mean values being −569.40 ± 37.78 HU for the right lung and −579.94 ± 34.21 HU for the left lung.

A paired *t*-test revealed a statistically significant difference in lung attenuation between the right and left lungs during sedation (*p* = 0.003). However, no statistically significant difference was found between the lungs during the spontaneous breathing under anesthesia. The difference became significant again during apnea (*p* = 0.032).

A high statistical difference was found in the paired *t*-test between the mean attenuation values registered during sedation, after anesthesia induction and following apnea, for the right (*p* = 0.005 and *p* < 0.001 respectively) and the left lung (*p* < 0.001). No statistical difference was found for mean attenuation values for the right and left lungs between anesthesia with spontaneous breathing and apnea.

Concerning aerated areas, the results showed highly significant differences in the attenuation values for both lungs in sedated animals compared to those during spontaneous breathing under anesthesia (*p* < 0.0001) and during induced apnea (*p* < 0.0001) (Table 1). No statistically significant differences were observed in attenuation values between spontaneous breathing under anesthesia and induced apnea for either lung.

For poorly aerated lung tissue (−499 to −101 HU), the mean lung attenuation for the right lung was −338.13 ± 8.18 HU in sedated animals, −335.65 ± 14.87 HU during spontaneous breathing under anesthesia, and −340.38 ± 12.67 HU during induced apnea. For the left lung, the mean attenuation values were −340.626 ± 9.50 HU, −335.48 ± 18.12 HU, and −341.41 ± 17.00 HU for sedated, spontaneous breathing, and apnea conditions, respectively. No statistically significant differences were observed in a paired *t*-test between the values obtained in the other three groups for poorly aerated lungs.

## 4. Discussion

This study aimed to identify the impact of sedation and anesthesia on lung volume and attenuation in CT scans. Both our initial research hypotheses were statistically confirmed; the results show that anesthesia impacts lung volume and attenuation, resulting in a marked decrease in total lung volume and an increase in attenuation. This is similar in both lungs and potentially impairs the accuracy of lung CT evaluations. These findings align with studies in other species, like dogs [17] and children [18].

Lung volume was calculated by means of threshold masking to select lung parenchyma in the detriment of nearby tissues [11,16,19]. An Intraclass Correlation Coefficient (ICC) ranging from 0.957 to 0.999 for repeatability, with the lower limits of the 95% confidence intervals (CI) being ≥0.85, indicates a strong level of agreement between the two measurement sessions. These high ICC values demonstrate that the measurements can be considered highly repeatable, reflecting consistent performance across repeated tests [20].

The Pulmonary Index obtained was slightly higher than the one found in 1977 by Boatman (15.28 cm^3^/kg against 17.79 cm^3^/kg), although the reported volume was found by casting the excised lungs [21]. The low R^2^ of 0.42 indicates a moderate positive correlation, meaning that there is a significant relationship; i.e., 42% of the lung volume is explained by the variation in weight, but it does not seem powerful enough to predict lung volume confidently based solely on body weight [21].

Computed tomography is considered the gold standard for the clinical evaluation of lung diseases in rabbits [5,6], but protocols for lung CT exams are lacking in the literature. In 2017, the study of Müllhaupt et al. [7] was the first to deliver information on normal lung volumes and attenuation in anesthetized rabbits. The mean volumes presented in that study for the right and left lungs were statistically different, with the right lung volume being larger than that of the left lung. These findings were consistent with our data. The lung volumes calculated in the previous study were not statistically significantly different from those obtained in our study when the animals were under anesthesia during spontaneous breathing and apnea. Although the anesthetic protocols were different, both produced surgical anesthesia, and the effects on the lung volume were found to be equal.

As expected, the dependent lung regions were more significantly affected by anesthesia, primarily due to the loss of diaphragmatic muscle tone. This reduction in muscle tone decreases the pressure gradient between the thoracic and abdominal cavities, leading to compression atelectasis [22]. The mean volume loss observed in the dependent lung areas was approximately 50%, while the non-dependent areas experienced a reduction of about 34%. In human medicine, it is estimated that anesthesia can cause a total lung volume decrease of around 20% [23].

A study on foals reported that sedation increased tidal volume by 28%, accompanied by a reduction in respiratory rate. However, subsequent anesthesia resulted in a 34% reduction in lung volume without a compensatory increase in respiratory rate [24]. These findings suggest that sedation may account for the larger lung volumes observed in sedated rabbits in our study compared to those under anesthesia. Future studies should investigate the differences in lung volumes between awake and sedated states to clarify this observation.

Mean lung attenuation is expected to rise rapidly after anesthesia induction and remain stable during extended periods [13,25,26,27]. In our study, the mean lung attenuation rose rapidly after anesthesia induction. Positive pressure ventilation has been used in clinical settings and experimentally to reduce atelectasis during lung CT scans and to induce apnea [24,28,29]. A trivial decrease in lung attenuation was found in our study after apnea induction, but it was not statistically different from the values obtained while spontaneously breathing during anesthesia. A study in cats showed that apnea induction by hyperventilation did not decrease the areas of atelectasis [30]; this finding can explain the trivial difference found in our study.

Mean lung attenuation for aerated tissue (−899 to −500 HU) was statistically different between sedation and anesthesia, but it was not different between spontaneous breathing and apnea. On the other hand, the mean attenuation of poorly aerated lungs did not show any statistically significant difference between groups. These means were calculated by the application of the selected thresholds to produce segmentation; this rendered the division of the parenchyma in aerated and poorly aerated areas, applying a bias to the results, and this was a limitation. On the other hand, global lung attenuation for the right and left lungs allowed us to compare our results with the previously published data, finding concordance between the mean lung attenuation registered for each lung during anesthesia and values published. These findings allow us to say that mean lung attenuation for rabbits’ right and left lungs under general anesthesia is expected to be close to the means in our study.

## 5. Conclusions

Our study demonstrates that sedated rabbits exhibit higher mean lung volumes and attenuation values on CT scans compared to those when they are anesthetized, even after undergoing hyperventilation. This suggests that sedation alone offers advantages in thoracic CT imaging by minimizing artifacts and distortions typically introduced by anesthesia. These artifacts can result from factors like reduced lung aeration and atelectasis caused by the loss of muscle tone during anesthesia. Consequently, sedation allows for a more accurate evaluation of lung structures and pathology.

Thus, our findings support the feasibility of using sedation as a practical alternative to general anesthesia when restraining rabbits during thoracic CT examinations. Sedation may be preferable in clinical practice for cases where the preservation of lung volume and accurate assessment of lung tissue attenuation are crucial, making it a potentially better choice for diagnostic imaging of the respiratory system in rabbits.

## Figures and Tables

**Table 1:** Repeatability of lung volume (cm^3^) and attenuation (Hounsfield Units) in sedation.

Variable	Session	Minimum	Maximum	Mean ± SD	Paired *t*-Test	ICC (95%CI)
Right lung volume	I	28.739	46.423	38.82 ± 5.11	0.994	0.96 (0.905–0.987)
	II	28.450	46.973	38.82 ± 4.95		
Left lung volume	I	22.456	39.974	30.57 ± 5.54	0.208	0.976 (0.936–0.991)
	II	21.289	40.085	30.97 ± 5.82		
Total lung volume	I	52.513	85.769	69.39 ± 10.04	0.060	0.998 (0.995–0.998)
	II	53.016	86.05	69.68 ± 9.89		
Aerated right lung attenuation	I	−698.944	−634.225	−675.08 ± 18.23	0.747	0.996 (0.99–0.999)
	II	−699.133	−636.572	−675.21 ± 18.37		
Aerated left lung attenuation	I	−708.961	−666.244	−690.03 ± 14.19	0.862	0.992 (0.978–0.997)
	II	−708.822	−664.883	−689.96 ± 13.96		

CI, confidence interval; ICC-Intraclass Correlation Coefficient; SD, standard deviation.

**Table 2:** Repeatability of lung volume (cm^3^) and attenuation (Hounsfield Units) in anesthesia.

Variable	Session	Minimum	Maximum	Mean ± SD	Paired *t*-Test	ICC (95%CI)
Total lung volume	I	18.952	35.261	27.82 ± 5.40	0.707	0.976 (0.934–0.991)
	II	19.164	35.276	27.70 ± 5.62		
Left lung volume	I	12.632	29.688	19.28 ± 4.41	0.200	0.979 (0.943–0.992)
	II	12.970	30.215	19.58 ± 4.56		
Total lung volume	I	32.861	64.832	47.10 ± 9.28	0.470	0.994 (0.983–0.998)
	II	33.441	65.491	47.29 ± 9.60		
Aerated right lung attenuation	I	−685.725	−578.192	−633.48 ± 36.15	0.069	0.999 (0.997–1.000)
	II	−685.825	−579.140	−632.73 ± 35.61		
Aerated left lung attenuation	I	−692.192	−574.591	−632.24 ± 38.75	0.124	0.998 (0.994–0.999)
	II	−691.120	−574.792	−631.20 ± 39.63		

CI, confidence interval; ICC-Intraclass Correlation Coefficient; SD, standard deviation.

**Table 3:** Repeatability of lung volume (cm^3^) and attenuation (Hounsfield Units) in apnea.

Variable	Session	Minimum	Maximum	Mean ± SD	Paired *t*-Test	ICC (95%CI)
Total lung volume	I	19.678	35.231	28.26 ± 3.88	0.916	0.923 (0.801–0.971)
	II	18.982	35.619	28.22 ± 4.56		
Left lung volume	I	13.258	30.957	20.34 ± 4.36	0.618	0.917 (0.787–0.969)
	II	14.754	31.021	20.13 ± 4.11		
Total lung volume	I	36.141	66.188	48.60 ± 7.40	0.614	0.964 (0.903–0.987)
	II	35.057	66.640	48.35 ± 7.81		
Aerated right lung attenuation	I	−676.742	−544.738	−627.23 ± 35.02	0.349	1.000 (0.999–1.000)
	II	−675.811	−544.447	−627.05 ± 35.02		
Aerated left lung attenuation	I	−696.140	−555.915	−632.77 ± 38.28	0.092	0.999 (0.999–1.000)
	II	−695.299	−552.714	−632.27 ± 38.59		

CI, confidence interval; ICC-Intraclass Correlation Coefficient; SD, standard deviation.

**Table 4:** Evaluation of inflated lung volumes (cm^3^) and attenuation (HU) using the segmentation (−899 to −500 HU).

Lung Region		Sedated		Anesthesia		Apnea	
		Volume	Attenuation	Volume	Attenuation	Volume	Attenuation
Dorsal	Right Lung	16.10 ± 2.53	−684.16 ± 19.62	10.37 ± 3.54	−644.60 ± 36.64	13.09 ± 3.14	−648.95 ± 34.56
	Left Lung	12.46 ± 2.38	−692.65 ± 16.67	7.41 ± 2.93	−657.67 ± 36	9.50 ± 2.66	−659.53 ± 34.86
Ventral	Right Lung	11.52 ± 2.57	−667.33 ± 18.35	5.50 ± 2.89	−624.74 ± 32.94	6.73 ± 2.54	−621.54 ± 34.56
	Left Lung	10.30 ± 2.26	−686.80 ± 18.39	4.80 ± 3.07	−632.61 ± 33.31	5.96 ± 2.50	−624.52 ± 28.93

## Data Availability

The raw data supporting the conclusions of this article will be made available by the authors on request.
